# In Vitro Bioelectrochemical Properties of Second-Generation Oxide Nanotubes on Ti–13Zr–13Nb Biomedical Alloy

**DOI:** 10.3390/ma16041408

**Published:** 2023-02-08

**Authors:** Agnieszka Stróż, Thomas Luxbacher, Karolina Dudek, Bartosz Chmiela, Patrycja Osak, Bożena Łosiewicz

**Affiliations:** 1 Faculty of Science and Technology, Institute of Materials Engineering, University of Silesia in Katowice, 75 Pułku Piechoty 1A, 41-500 Chorzów, Poland; 2 Anton Paar GmbH, Street 20, 8054 Graz, Austria; 3 Refractory Materials Center, Institute of Ceramics and Building Materials, Łukasiewicz Research Network, Toszecka 99, 44-100 Gliwice, Poland; 4 Insitute of Materials Science, Silesian University of Technology, Z. Krasińskiego 8, 40-019 Katowice, Poland

**Keywords:** anodizing, corrosion resistance, oxide nanotubes, Ti–13Zr–13Nb alloy, zeta potential

## Abstract

Surface charge and in vitro corrosion resistance are some of the key parameters characterizing biomaterials in the interaction of the implant with the biological environment. Hence, this work investigates the in vitro bioelectrochemical behavior of newly developed oxide nanotubes (ONTs) layers of second-generation (2G) on a Ti–13Zr–13Nb alloy. The 2G ONTs were produced by anodization in 1 M (NH_4_)_2_SO_4_ solution with 2 wt.% of NH_4_F. The physical and chemical properties of the obtained bamboo-inspired 2G ONTs were characterized using scanning electron microscopy with field emission and energy dispersive spectroscopy. Zeta potential measurements for the examined materials were carried out using an electrokinetic analyzer in aqueous electrolytes of potassium chloride, phosphate-buffered saline and artificial blood. It was found that the electrolyte type and the ionic strength affect the bioelectrochemical properties of 2G ONTs layers. Open circuit potential and anodic polarization curve results proved the influence of anodizing on the improvement of in vitro corrosion resistance of the Ti–13Zr–13Nb alloy in PBS solution. The anodizing conditions used can be proposed for the production of long-term implants, which are not susceptible to pitting corrosion up to 9.4 V.

## 1. Introduction

Innovative biomaterials inspired by nature are the answer to the key challenges of modern medicine. Current scientific trends in medicine concern the use of intelligent bionanomaterials for the needs of dynamically developing regenerative medicine, tissue engineering and targeted therapy [1,2,3]. The latest generation of bionanomaterials that can give hope to hundreds of thousands of patients waiting in queues for their health and life include oxide nanotubes (ONTs) inspired by the structure of bamboo obtained on titanium and titanium alloys intended for long-term implants [4,5,6,7,8,9,10,11,12,13,14,15,16,17,18,19,20,21]. Currently, titanium and its alloys are commonly used metallic biomaterials in medicine due to their unique properties, which include high corrosion resistance [1,4,5,6,11,15,20,22,23,24,25,26,27], biological inertness [1,11,28], low specific gravity and excellent mechanical properties [1,7,16,24,29,30,31,32,33,34]. In vitro corrosion resistance of metallic biomaterials affects their functionality and durability. It is also the main factor that determines biocompatibility. According to the fundamental paradigm of metallic biomaterials, which does not apply only to biodegradable metals, the more corrosion-resistant a biomaterial is, the greater its biocompatibility [27]. The Ti–13Zr–13Nb alloy belongs to the newest group of titanium alloys, which do not contain allergenic nickel or aluminum and carcinogenic vanadium [4,5,11,12,13,14,15,18,19,23,32,33,34,35]. This bi-phase (α + β) alloy shows high biotolerance and very good corrosion resistance and is classified as a long-lasting biomaterial [4,5,11,12,13,14,15,18,19,23,32,34]. The biocompatibility of titanium and its alloys results from the presence of a native oxide layer on its surface, which is characterized by thermodynamic stability and low electronic conductivity. Its thickness ranges from 2 to 10 nm and provides high corrosion resistance [23]. In the biological environment of the human body, in the oxide layer on the surface of titanium and its alloys, processes involving the incorporation of elements from the fluids of cells and tissues surrounding the implant takes place. Moreover, an in vitro hemocompatibility study of the Ti–13Zr–13Nb alloy before and after anodizing in 1 M ethylene glycol solution with 4 wt.% of NH_4_F revealed the hemolytic index of 0.30 (8) and 0.00 before and after surface modification, respectively [11]. The obtained results confirmed that anodizing of the Ti–13Zr–13Nb biomedical alloy allowed the complete elimination of hemolysis.

To increase the biocompatibility of the Ti–13Zr–13Nb alloy and improve its biological activity, we propose modifying the surface of this alloy using the anodizing method, making it possible to produce self-organized ONTs of various geometry and length. Bamboo-like ONTs layers were obtained on the Ti–13Zr–3Nb alloy using inorganic and organic electrolytes [4,5,12,13,14,15,18,19]. The porous layers of ONTs can be additionally modified by enriching them with electrolyte components, e.g., phosphates, giving them bioactivity features. The ONTs can also be saturated with therapeutic agents, bactericides, active substances or tissue-forming hydroxyapatite, thanks to which they can be used in orthopedics, dentistry and intelligent drug delivery systems [10]. The bamboo-like ONTs enable the strengthening of bone functions at the boundary of the implant and bones. In the case of the rough surface of titanium implants, an increase in osteogenic properties such as cell proliferation, protein adsorption and deposition of calcium have been reported, which favor osseointegration [1,2,3].

As a result of surface modification, the physical properties change, which also affects the chemical properties of the surface. The implant affects the surrounding tissues through its surface. This is due to the interaction of implant surfaces and body fluids, which is often mediated by adsorbed proteins [5,11,20,22,23,24,25,26]. The features of the implant surface, regarding its roughness, topography and surface chemistry, are then “translated” by the protein layer into information that is understandable by the cells.

A sensitive indicator for the actual surface charge of a biomaterial in contact with a biological environment is the zeta potential (ζ) [36,37,38]. Wettability and surface roughness of ONTs formed on the Ti–13Zr–13Nb alloy implant surfaces are characterized in the literature, while the surface charge is still unavailable [39,40]. Studying the zeta potential of the biocompatible Ti–13Zr–13Nb alloy before electrochemical modification and with vertically oriented ONTs will allow learning the mechanism by which ions will adhere better to smooth surfaces and will prefer a porous structure. The technique of streaming potential is based on the phenomenon of creating an electric field when the electrolyte flows, which remains tangential to the stationary, charged surface of the tested material. The ζ value is calculated from the generated streaming potential. The local surface charge is related to the surface roughness of ONTs in the nanoscale. On the edges of ONTs with different internal and external diameters, there is a high surface charge density, which is the binding site for monovalent and divalent ions, as well as proteins mediating osteoblast adhesion [39,40]. Despite this, there is a lack of data in the literature regarding this important bioelectrochemical property of charged solid–liquid interfaces for the Ti–13Zr–13Nb alloy in body fluids. Therefore, the main purpose of this work was an evaluation of the anodizing effect on ζ and in vitro corrosion resistance of the Ti–13Zr–13Nb alloy in artificial body fluid. This work brings a new contribution to the description of the relationship between the new anodizing conditions and in vitro bioelectrochemical properties of the latest generation Ti–13Zr–13Nb alloy, which has been intensively researched in recent years due to its unique properties.

## 2. Materials and Methods

### 2.1. Substrate Treatment

The material under study was Ti–13Zr–13Nb (wt.%) alloy (BIMO TECH, Wrocław, Poland). Disc-shaped samples with a thickness of 5 mm were cut from a wire with a diameter of 20 mm and a length of 1 m. A specification covering chemical, mechanical and metallurgical requirements for wrought Ti–13Nb–13Zr alloy for surgical implant applications is provided in standard ASTM F1713-08(2021)e1 [41]. The samples were subjected to one-sided wet grinding on a metallographic grinding and polishing machine Forcipol 202 (Metkon Instruments Inc., Bursa, Turkey) at 250 rpm of the grinding wheel with soft start and soft stop. SiC abrasive papers of P600, P1200 and P3000 gradations (Buehler Ltd., Lake Bluff, IL, USA) were used. The ground samples with a mirror-like surface were rinsed thoroughly under tap water and sonicated for 20 min in acetone (Avantor Performance Materials Poland S.A., Gliwice, Poland) and then in ultrapure water with resistivity of 18.2 MΩ cm (Milli-Q Advantage A10 Water Purification System, Millipore SAS, Molsheim, France). The cleaning procedure in ultrapure water was repeated twice with a change of water.

### 2.2. Production of ONTs on Ti–13Zr–13Nb Alloy

A detailed method of preparing anodes was described in our previous work [10]. The self-passive oxide layer on the anode surface was removed immediately before electrochemical oxidation by dissolving in 25% *v*/*v* HNO_3_ (Avantor Performance Materials Poland S.A., Gliwice, Poland) for 10 min at room temperature. The depassivated anodes were cleaned with Milli-Q water in an ultrasonic bath for 20 min. 

To produce the ONTs layers, the prepared anodes were subjected to one-step anodizing. Electrochemical oxidation was carried out in 1 M (NH_4_)_2_SO_4_ solution with 2 wt.% of NH_4_F at 20 V for 120 min at room temperature. Ammonium sulfate (≥99.0% purity) and ammonium fluoride (≥99.99% trace metals basis) were supplied by Sigma-Aldrich (Saint Louis, MI, USA). The mechanism of obtaining 2G ONTs layers on the surface of the Ti–13Zr–13Nb alloy under the applied anodizing conditions was described in detail in our earlier work [13]. Anodizing was conducted in a two-electrode system in which the anode was a sample tested in a Teflon holder, while the 4 cm^2^ platinum mesh served as a counter electrode. The geometric surface of the anode subjected to electrochemical oxidation was 0.64 cm^2^. The distance between the cathode and anode was 25 mm. After anodizing, each anode was immersed for 5 min in Milli-Q water subjected to vigorous agitation.

### 2.3. Physicochemical Characteristics of ONTs on Ti–13Zr–13Nb Alloy

Surface morphology and thickness of 2G ONTs layers obtained on the Ti–13Zr–13Nb alloy were examined using a scanning electron microscope with field emission (FE-SEM) Hitachi HD-2300A (Hitachi Ltd., Tokyo, Japan) under low-vacuum conditions of 50 Pa at an accelerating voltage of 15 kV. FE-SEM images were collected by secondary electrons (SE). Before microscopic examinations, a 5 nm chromium layer was deposited on the surface of the tested samples using an ion sputtering machine Quorum Q150T ES equipment (Quorum Technologies, East Sussex, UK) with argon as the ion extracting source. Local chemical composition with the surface distribution of elements was performed using an Energy Dispersive Spectrometer (EDS, Oxford Instruments, Abingdon, UK).

### 2.4. In Vitro Surface Characteristics of ONTs on Ti–13Zr–13Nb Alloy in Body Fluids 

**Figure 1:**
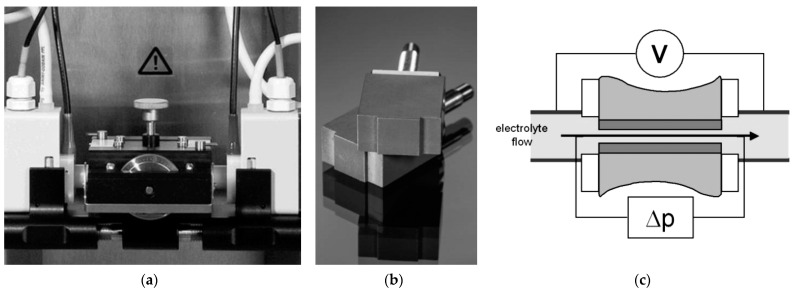
Adjustable Gap Cell mounted between electrodes (**a**); Sample holder (10 × 10 mm, center) (**b**); Measuring principle (**c**).

The surface zeta potential for the non-anodized and anodized Ti–13Zr–13Nb alloy was measured in aqueous electrolytes with different ionic strengths. The following solutions were applied: KCl (0.001 mol L^−1^) as the background electrolyte, PBS (0.001 mol L^−l^ and 0.01 mol L^−l^) [42] and artificial blood (0.01 mol L^−l^) [43] in a wide pH range from over 3 to 9 at 37(2) °C. The pH of these aqueous electrolytes was adjusted with 0.05 mol L^−1^ HCl and 0.05 mol L^−l^ NaOH, respectively. Streaming current measurements were performed with an electrokinetic analyzer SurPASS 3 (Anton Paar GmbH, Graz, Austria) for surface testing of materials with automatic ζ analysis. The Adjustable Gap Cell shown in Figure 1a was used. During the measurement of ζ, a pair of the same samples were used, which were fixed in the holders with a cross-section of 10 × 10 mm using double-sided adhesive tape (Figure 1b). The sample holders were inserted into the Adjustable Gap Cell in such a way that the tested surfaces of the discs faced each other. Both tested surfaces were located at a distance of about 100 μm. The SurPASS 3 instrument enabled the use of both the classic streaming potential method and the streaming current method for direct analysis of the surface zeta potential. The surface conductance of the investigated sample influenced the surface zeta potential of tested surfaces evaluated from streaming potential measurements. In turn, the streaming current method required that the geometry of the flow channel be known (Figure 1c). The ζ of planar samples was preferably determined from the measurement of the streaming current due to their electrical conductivity.

**Figure 2:**
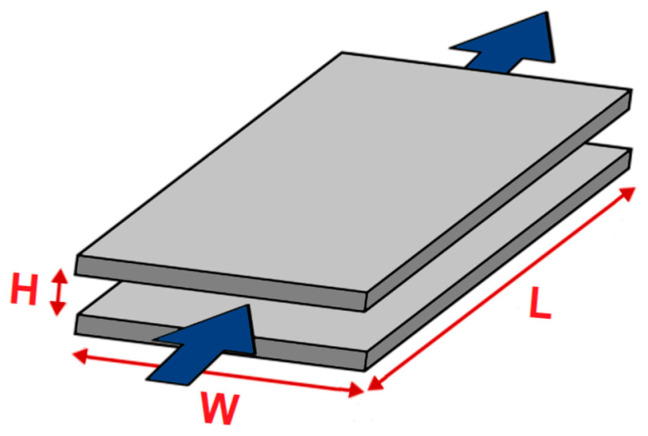
Scheme of the rectangular slit channel between adjacent solid samples with a planar surface. L, W and H are the length, width and height of the flow channel, respectively.

Scheme of the rectangular slit channel between adjacent solid samples with a planar surface indicating its dimensions is presented in Figure 2. L, W and H stand for the length, width and height of the flow channel, respectively. The blue arrows indicate the direction of electrolyte flow during the ζ measurement.

The fundamental Helmholtz–Smoluchowski dependence for evaluating the ζ from streaming current measurements is given by Equation (1), which relates electrokinetic phenomena like the streaming potential and the streaming current to the zeta potential [39,40]:

(1) $$\zeta =\frac{{\mathrm{dI}}_{\mathrm{str}}}{d\Delta p}\cdot \frac{\eta }{\varepsilon_{\mathrm{rel}}\cdot \varepsilon_{0}}\cdot \frac{L}{A}$$

In Equation (1), the measured streaming current coupling coefficient dIstr/dΔp is related to the cell constant L/A of the flow channel, which is the gap between adjacent solid samples. As shown schematically in Figure 2, L denotes the length of the rectangular slit channel formed between two planar surfaces and A is its cross-section, A = W × H, with W denoting channel width and H gap height. In Equation (1), η is the viscosity of the electrolyte, ε_rel_ is related to the dielectric coefficient of the electrolyte and ε_0_ denotes the vacuum permittivity. For dilute aqueous solutions, η and ε_rel_ of water were used. The application of Equation (1) requires a thorough knowledge of the flow channel geometry, defined by the L/A cell constant. In case of the rectangular slot channel, the L and W parameters are determined by the size of the solid sample. The value of the parameter H, which is the gap height, can be calculated based on the measured volume flow rate of electrolyte passing through the flow channel driven by the applied differential pressure. 

### 2.5. In Vitro Corrosion Resistance of ONTs on Ti–13Zr–13Nb Alloy in PBS

In vitro corrosion resistance measurements of the non-anodized Ti–13Zr–13Nb alloy with 2G ONTs layers were conducted in PBS at 37(2) °C using the method of open circuit potential (OCP) and anodic polarization curves. For the preparation of PBS containing 8.0 g L^−1^ NaCl, 0.2 g L^−1^ KCl, 1.42 g L^−1^ Na_2_HPO_4_ and 0.24 g L^−1^ KH_2_PO_4_ [42] analytically pure reagents (Avantor Performance Materials Poland S.A., Gliwice, Poland) and Milli-Q water were used. The pH of the PBS solution was adjusted to 7.4(1) using 4% NaOH and 1% C_3_H_6_O_3_. Prior to each measurement, a fresh portion of PBS was deaerated using an Ar flow (UHP Ar, 99.999%) for 20 min. Corrosion behavior of the tested electrodes was characterized in a single-chamber electrochemical cell using a conventional three-electrode system. The cathode was the Ti–13Zr–13Nb alloy without and with the ONTs layer. The anode was platinum foil with dimensions of 40 × 20 × 2 mm. The reference electrode was the saturated calomel electrode (SCE) immersed in PBS electrolyte using Luggin capillary. Open circuit potential (E_OC_) was stabilized for 2 h. Then, the anodic polarization curves in the range of potentials from E_OC_ minus 150 mV to 9.4 V were registered at the polarization rate of v = 1 mV s^−1^. All electrochemical tests were conducted using the Autolab/PGSTAT20 computer-controlled electrochemical system (Metrohm Autolab B.V., Utrecht, The Netherlands) equipped with the General Purpose Electrochemical System software.

## 3. Results and Discussion

### 3.1. FE-SEM/EDS Studies of ONTs on Ti–13Zr–13Nb Alloy

**Figure 3:**
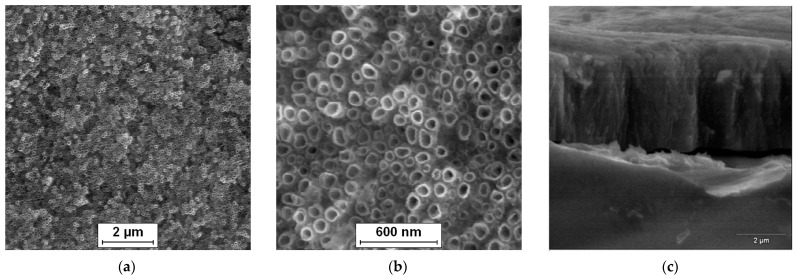
SE FE-SEM image of the Ti–13Zr–13Nb alloy after anodizing in 1 M (NH_4_)_2_SO_4_ solution with 1 wt.% of NH_4_F at 20 V for 120 min: (**a**) On-top general view of 2G ONTs layer; (**b**) View of 2G ONTs layer in a selected micro-region; (**c**) Fracture of 2G ONTs layer [14].

The surface morphology of the Ti–13Zr–13Nb alloy after anodizing in 1 M (NH_4_)_2_SO_4_ solution with 2 wt.% of NH_4_F at 20 V for 120 min can be observed in Figure 3. The SE FE-SEM image in Figure 3a presents the on-top general view of the 2G ONTs layer. Figure 3b shows the surface morphology of the obtained bamboo-like ONTs in more detail. Oxide nanotubes with a circular cross-section and single walls are evenly distributed over the observed surface and arranged vertically. Bundles of ONTs that grew in local areas corresponding to the presence of α and β phases are also visible [15]. The multi-step process of 2G ONTs layer formation on the surface of the Ti–13Zr–13Nb alloy in aqueous solutions containing fluoride ions was discussed in detail in our earlier work [13].

Figure 3c presents an exemplary SE FE-SEM image of the mechanically scratched region of the 2G ONTs layer formed on the Ti–13Zr–13Nb substrate. The top view of the mechanically fractured oxide layer reveals micro-areas with invisible and visible bamboo-like nanotubes, which form an ordered matrix of vertically oriented ONTs. The high degree of ordering of smooth-walled ONTs may increase the corrosion resistance of the Ti–13Zr–13Nb alloy in body fluids [4,5,11]. The inner diameter, outer diameter and length of the obtained 2G ONTs layer were determined based on the FE-SEM images recorded from the selected areas of the Ti–13Zr–13Nb alloy surface in our preliminary results [14]. Empirical distribution histograms of the ONTs’ diameters allowed determining the average values of the morphological parameters. It was found that under the proposed conditions, bamboo-like ONTs with an inner diameter of 61(11) nm and an outer diameter of 103(16) nm were obtained. The average value of 2G ONTs length was 3.9(2) µm, and the specific surface area per cm^2^ was equal to 15.6 cm^2^ cm^−2^. 

**Figure 4:**
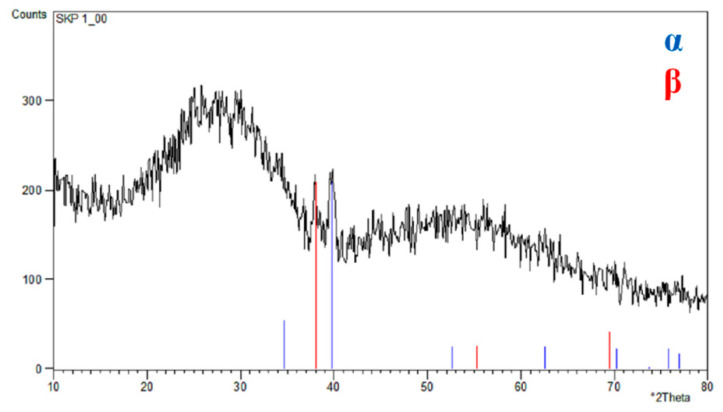
GIXD pattern of Ti–13Zr–13Nb after anodization in 1 M (NH_4_)_2_SO_4_ with 2 wt.% NH_4_F [14].

The X-ray structural investigations of the 2G ONTs layer obtained in 1 M NH_4_(SO_4_)_2_ solution with 2 wt.% NH_4_F on the Ti–13Zr–13Nb alloy surface were carried out in the previous work [14]. The grazing incidence X-ray diffraction (GIXD) results shown in Figure 4 revealed the presence of α-Ti and β-Ti phases for the bi-phase Ti–13Zr–13Nb alloy substrate and an amorphous halo related to the 2G ONTs layer.

**Figure 5:**
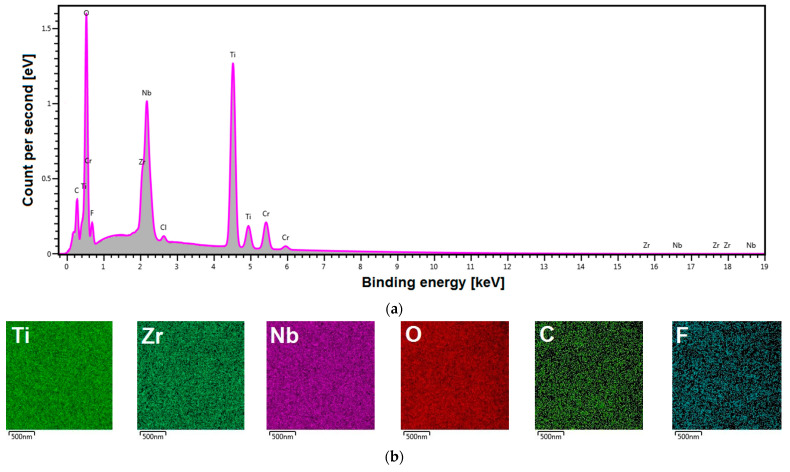
Local chemical composition of the Ti–13Zr–13Nb alloy with the 2G ONTs layer: (**a**) Energy dispersive spectrum in the micro-region; (**b**) EDS maps of elements distribution (Ti, Zr, Nb, O, C, F) in the micro-region.

A new generation of biomedical titanium alloy was selected for research in this work. By eliminating toxic elements such as vanadium, aluminum and nickel from the composition of the titanium alloy, the appropriate requirements for medical implant applications were ensured [32]. The chemical composition of the Ti–13Zr–13Nb alloy was investigated in our previous work [15]. The results of the analysis of the local chemical composition of the Ti–13Zr–13Nb alloy with the obtained 2G ONTs layer are shown in Figure 5. 

A representative energy dispersive spectrum in the micro-region on the surface of the anodized Ti–13Zr–13Nb alloy shows the relationship between the count per second and the binding energy (Figure 5a). Peaks from Ti, Zr and Nb alloying elements are visible. The presence of an oxygen-derived peak of high intensity in the EDS spectrum in Figure 5a testifies that, on the surface of the tested biomaterial, an oxide layer is present. The obtained EDS spectrum also reveals the trace amounts of the elements included in the electrolyte used for anodizing (F, Cl), an ultra-thin layer applied to improve the conductivity of the tested sample (Cr) or impurities (C).

Figure 5b show the corresponding distribution maps of chemical elements in the micro-region. EDS distribution maps for individual elements have been recorded in different colors, which allows distinguishing the location of elements such as Ti, Zr, Nb, O, C and F. The obtained EDS distribution maps show that all identified elements are evenly distributed on the surface in the studied micro-region, and C and F occur in small amounts. 

### 3.2. In Vitro Bioelectrochemical Characteristics in Body Fluids 

**Figure 6:**
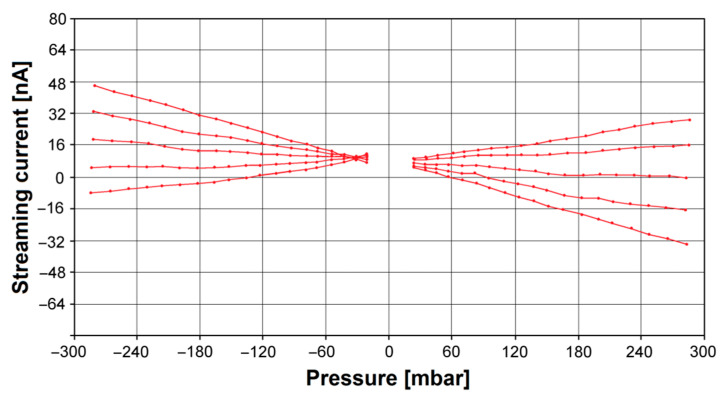
Streaming current vs. differential pressure for the Ti–13Zr–13Nb alloy after anodizing at various pH of the electrolyte.

The zeta potential is related to the surface charge at the biomaterial | electrolyte interface, and its knowledge allows for characterizing surface properties and designing new biomaterials. To determine the ζ parameter for the Ti–13Zr–13Nb alloy before and after anodizing, the measurement of streaming current was performed alternatively in both flow directions. Figure 6 shows exemplary pressure ramps as streaming current vs. differential pressure for the Ti–13Zr–13Nb alloy after anodizing at different pH of the electrolyte. One can see that the dependence representing the streaming current on the applied differential pressure is strictly linear, and the linear regression coefficients take values higher than R^2^ = 0.99. 

**Figure 7:**
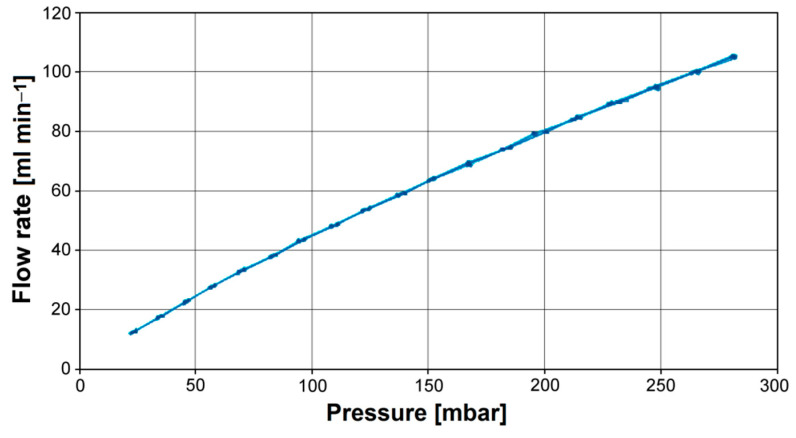
Flow rate vs. differential pressure for the Ti–13Zr–13Nb alloy after anodizing.

Figure 7 shows the flow behavior of electrolytes passing through the gap between exemplary sample surfaces for the Ti–13Zr–13Nb alloy after anodizing expressed as volume flow rate vs. differential pressure. For all series of measurements, a linear dependence that indicated laminar flow behavior was found.

**Figure 8:**
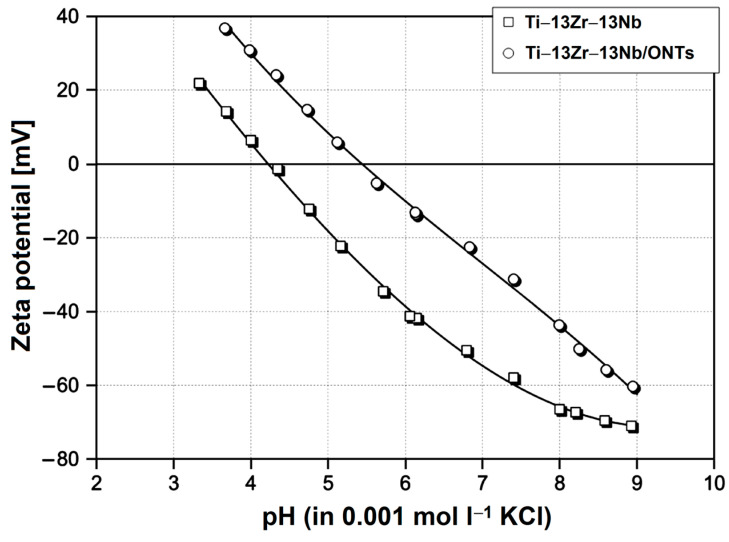
Dependence of electrolyte pH on zeta potential for the Ti–13Zr–13Nb alloy without and with the ONTs layer in 0.001 mol l^−1^ KCl.

The determined zeta potential for the Ti–13Zr–13Nb alloy with and without the ONTs layer in the presence of the inert aqueous solution of 0.001 mol L^−1^ KCl was compared. Figure 8 shows the dependence of electrolyte pH in the range of pH 3–9 on zeta potential, where the corresponding isoelectric points (IEPs) were found. Note that the pH was first changed to low pH by starting close to pH 6 (the native pH of a freshly prepared KCl solution) and adding acid. 

The measuring cell was then rinsed with Milli-Q water, thereby keeping the Ti–13Zr–13Nb alloy disks mounted. Afterward, the electrolyte was exchanged, and the titration proceeded to high pH. Note the coincidence of repetitive measurements close to pH 6, which indicates the stability of the Ti–13Zr–13Nb alloy surfaces in the presence of the aqueous solution in the pH range investigated. For the polished Ti–13Zr–13Nb alloy disks, a pH dependence on zeta potential with an IEP 4.2 typical for surfaces with little or no functional groups was found [44]. We assumed a native oxide layer present on the Ti–13Zr–13Nb substrate whose thickness was in the nanometer range [23]. This behavior was also found, e.g., for gold, stainless steel or polymer surfaces [44]. The growth of the ONTs on the Ti–13Zr–13Nb substrate is approved by the significant shift of the IEP to pH 5.4 and renders the surface of the anodized sample amphoteric. It was revealed that the 2G ONTs on the Ti–13Zr–13Nb substrate represent the amorphous oxide phase [14].

**Figure 9:**
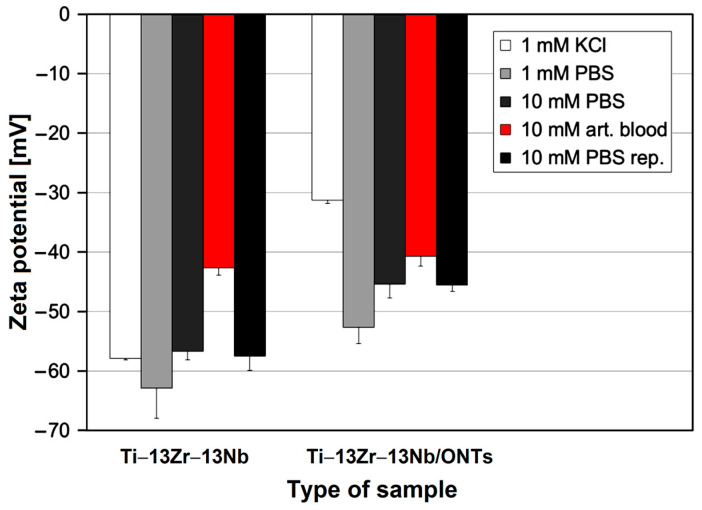
Zeta potential at pH 7.4 for the Ti–13Zr–13Nb alloy before and after anodizing in the presence of different buffer solutions, where the number of experiments for the error bars was n = 3.

In the next step, the effect of different buffer solutions on the zeta potential of the Ti–13Zr–13Nb alloy before and after anodizing was compared. Figure 9 shows the zeta potential at pH 7.4 for the tested materials in the presence of 0.001 mol L^−1^ KCl, 0.001 mol L^−1^ PBS, 0.01 mol L^−1^ PBS and 0.01 mol L^−1^ artificial blood, respectively. When exchanging the inert 1:1 electrolyte by PBS with comparable ionic strength, a significant increase in the negative ζ for the Ti–13Zr–13Nb/ONTs sample while the zeta potential remains almost unaffected for the Ti–13Zr–13Nb sample within the experimental error was found. We thus assumed a strong interaction of phosphate ions with the amorphous oxide surface. 

By further increasing the ionic strength of PBS to 0.01 mol L^−1^, a decrease in the zeta potential for samples Ti–13Zr–13Nb and Ti–13Zr–13Nb/ONTs was noted, which follows the prediction of the model of the double layer. Increasing ionic strength compresses the diffuse layer of surface-charge compensation counter-ions and thus reduces the magnitude of the zeta potential. This prediction assumes the absence of selective interaction between the electrolyte ions with the solid surface. An explanation for this observation is simply a smaller slope of the dependence of (negative) zeta potential on the ionic strength [44]. However, when exchanging the PBS buffer with artificial blood, thereby maintaining the ionic strength, a decrease in the negative zeta potential for all samples was found. This decrease is higher for the Ti–13Zr–13Nb sample (25%) as compared to the sample Ti–13Zr–13Nb/ONTs (10%). It was concluded that the complex ions contained in artificial blood have a stronger affinity to hydrophobic surfaces than to more hydrophilic ones. 

After completing the analysis in artificial blood, the disk samples were kept mounted in the Adjustable Gap Cell and rinsed with Milli-Q water. The repetitive measurement in the presence of 0.01 mol L^−1^ PBS for samples Ti–13Zr–13Nb and Ti–13Zr–13Nb/ONTs confirmed that the adsorption of complex ions contained in artificial blood was fully reversible. 

**Figure 10:**
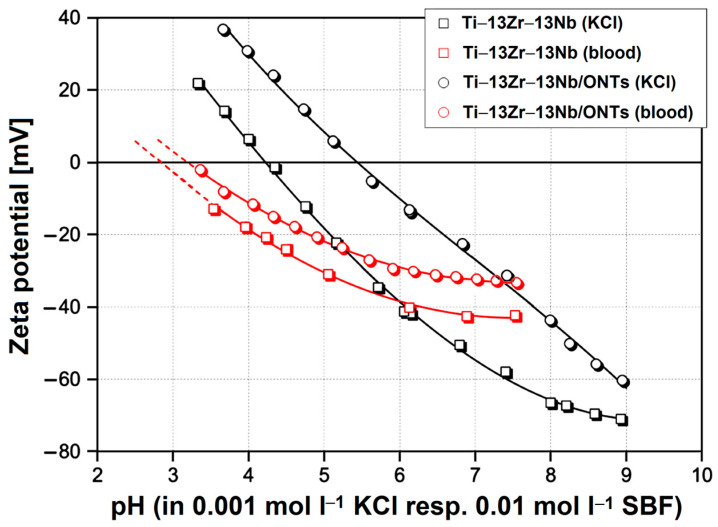
Dependence of electrolyte pH on zeta potential for the Ti–13Zr–13Nb alloy without and with the ONTs layer in 0.001 mol L^−1^ KCl and 0.01 mol L^−1^ simulated body fluid (SBF).

Since the zeta potential at pH 7.4 was negative for all samples and to determine the IEP, as a final measurement step, another titration starting at physiological pH and proceeding towards low pH was performed. Figure 10 shows the results, which confirm that the complex ions contained in the simulated body fluid (SBF) adsorb on the Ti–13Zr–13Nb alloy before and after anodizing and shift the IEP to low pH.

It is interesting to note that the effect of SBF is smaller for unoxidized Ti–13Zr–13Nb alloy. After finishing the pH titration, the sample disks were rinsed with Milli-Q water, and measurements were repeated for both types of samples in the presence of 0.01 mol L^−1^ PBS in order to investigate whether the adsorption of complex ions was reversible or permanent. The corresponding zeta potential at pH 7.4 is shown in Figure 9.

### 3.3. In Vitro Open Circuit Potential Characteristics in Body Fluids

**Figure 11:**
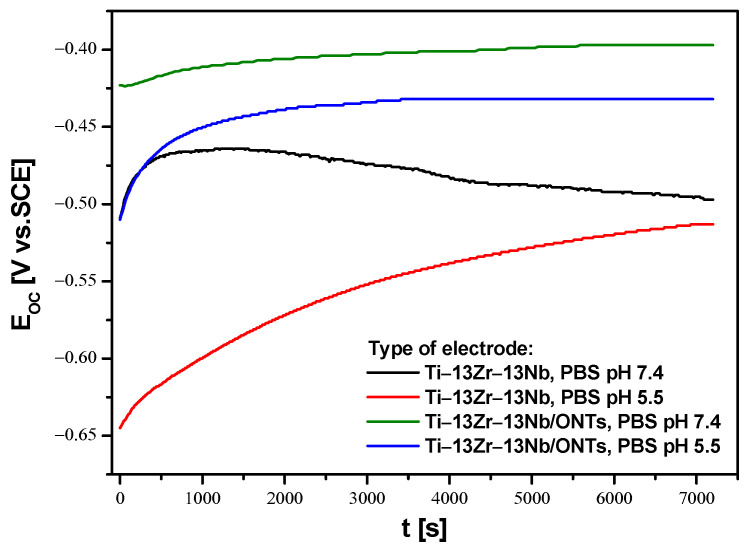
Open circuit potential (E_OC_) in the function of immersion time (t) for the Ti–13Zr–13Nb electrode without and with 2G ONTs layer in PBS at 37 °C.

The open circuit potential (E_OC_) parameter was used to determine the initial in vitro corrosion resistance of the Ti–13Zr–13Nb electrode before and after anodizing under conditions similar to those in the human body. Due to the fact that inflammation occurs immediately after implantation, which is associated with a decrease in pH in the acidic direction in the tissues surrounding the implant, electrochemical E_OC_ measurements were carried out in the PBS electrolyte at physiological and acidic pH. Changes in pH affect the risk of reduced corrosion resistance of the implant. Therefore, the OCP method was used to assess the protective properties of the obtained 2G ONTs layers on the Ti–13Zr–13Nb alloy. Figure 11 shows the course of E_OC_ for the Ti–13Zr–13Nb electrode without and with 2G ONTs layer for 2 h of immersion in PBC with pH 7.4 and 5.5 at 37 °C.

The ionic–electron equilibrium at the interfacial boundary between the electrode surface and electrolyte was reached after 7200 s of immersion. It should be noted that E_OC_ stabilized more slowly on the non-oxidized Ti–13Zr–13Nb electrode in both neutral and acidic PBS. This phenomenon is probably related to the self-passivation of the Ti–13Zr–13Nb electrode surface after immersion in the electrolyte and the thickening of the ultrathin oxide film [23]. In the presence of 2G ONTs layers with protective properties, E_OC_ stabilization occurred faster. The stabilized E_OC_ value, treated in further studies as an approximate value of corrosion potential (E_cor_), was negative for all tested electrodes. The non-anodized Ti–13Zr–13Nb electrode showed lower corrosion resistance in PBS in comparison to the Ti–13Zr–13Nb/ONTs electrode in both neutral and acidic electrolytes. Such changes in the E_OC_ indicate a decrement in the thermodynamic tendency to the corrosion of anodized Ti–13Zr–13Nb electrode. The lowest corrosion resistance was demonstrated by the non-anodized Ti–13Zr–13Nb electrode in PBS of pH 5.5, for which the average value of E_OC_ was −0.513(30) V. It means that the increase in the content of aggressive chloride ions in the electrolyte accelerated the corrosion processes. The highest average E_OC_ of −0.397(19) V was determined for the Ti–13Zr–13Nb/ONTs electrode in PBS of pH 7.4, which indicates that the obtained 2G ONTs layer has stronger barrier properties as compared to the native oxide layer. These results confirm that the application of the anodizing process under the conditions used can significantly improve the corrosion resistance of the Ti–13Zr–13Nb electrode in body fluids. 

### 3.4. In Vitro Susceptibility to Pitting Corrosion in Body Fluids

**Figure 12:**
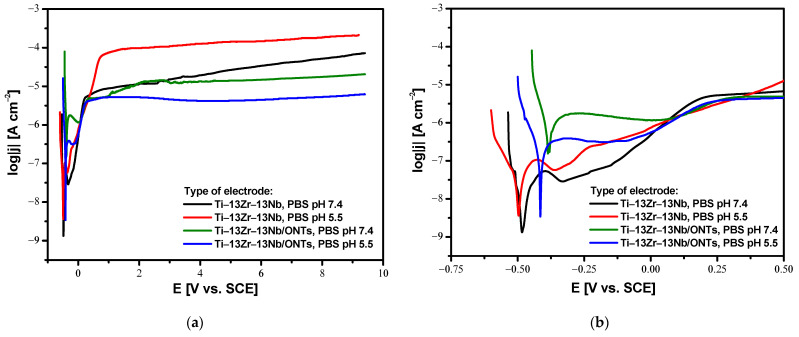
Anodic polarization curves for the Ti–13Zr–13Nb electrode without and with 2G ONTs layer in physiological and acidic PBS at 37 °C: (**a**) In the whole range of tested potentials; (**b**) The inset in the range of potentials corresponding to the cathode-anode transition.

Analysis of the anodic polarization curves shown in the semi-log form in Figure 12 revealed a similar course for all investigated electrodes with apparent passive anodic behavior. A shift towards cathode potentials for both Ti–13Zr–13Nb and Ti–13Zr–13Nb/ONTs electrodes in acidic PBS is observed as compared to physiological PBS. The reason for this is a decrease in the corrosion resistance caused by an increase in the aggressiveness of the corrosive environment. On the other hand, it can be seen that the production of the 2G ONTs layers by anodizing caused the desired shift of the log|j| = f(E) curves towards anodic potentials in both physiological and acid PBS in comparison with the non-anodized Ti–13Zr–13Nb electrode. In the range of potentials corresponding to the cathode branch with values lower than E_cor_, the tested electrodes are resistant to corrosion. At potentials equal to E_cor_, oxidation processes begin at the anode branch. The higher passive current densities of the order of 10^−4^ A cm^−2^ were observed for the Ti–13Zr–13Nb electrode in PBS of pH 5.5, which means the highest corrosion rate among the tested electrodes. The decrease in anodic current densities for the anodized Ti–13Zr–13Nb electrode in both PBS of pH 7.4 and 5.5 is associated with the presence of 2G ONTs layers which are characterized by greater stability as compared to the self-passive oxide layer on the Ti–13Zr–13Nb electrode surface.

It is worth noting that the Ti–13Zr–13Nb alloy, both before and after anodizing, did not show susceptibility to pitting corrosion because, in the range of the tested potentials up to 9.4 V, there was no breakdown of the oxide layers to the substrate. Registration of anode polarization curves for potentials above 9.4 V was impossible due to the limitations of the apparatus. The obtained results prove the excellent corrosion resistance of the tested biomaterials in environments containing chlorides, which can be proposed for the production of long-term implants. Compared to conventional steel biomaterials, the Ti–13Zr–13Nb alloy, both before and after anodizing, shows exceptional electrochemical properties in PBS solution [42]. An anodic behavior study of different types of stainless steel in PBS solution revealed that drastically lower breakdown potential (E_bd_) values were observed. At the E_bd_ of 0.208 V for AISI 304L and 0.559 V for AISI 316L austenitic stainless steels, as well as 1.120 V for 2205 duplex stainless steel, a sharp increase in current density on anodic polarization curves was observed, indicating much easier destruction of the oxide layer.

## 4. Conclusions

The obtained results confirm that the proposed conditions of anodizing for Ti–13Zr–13Nb alloy allow for obtaining bamboo-like 2G ONTs layers. It was found that the effect of electrolyte pH on the zeta potential of the examined surfaces occurred. In a neutral aqueous KCl solution, the 2G ONTs layer moves the isoelectric point from 4.2 for the non-anodized Ti–13Zr–13Nb alloy, which is typical for the surface without a functional group to pH of 5.4, which is characteristic for amorphous oxide phase. Comparison of the influence of different electrolytes such as KCl, PBS and artificial blood on the zeta potential at pH of 7.4 for the Ti–13Zr–13Nb alloy before and after anodizing revealed a strong reaction of calcium anions with amorphous surfaces. The complex ions contained in artificial blood have demonstrated a stronger affinity to the hydrophobic surface before anodizing than the hydrophilic one after electrochemical oxidation. The increase in corrosion resistance of the anodized Ti–13Zr–13Nb electrode in PBS as compared with the non-anodized Ti–13Zr–13Nb electrode was due to the presence of a stable 2G ONTs layer. For both types of electrodes, no susceptibility to pitting corrosion up to 9.4 V was found in potentiodynamic studies.

Knowledge of the surface charge of the biomaterial is of fundamental importance for predicting the biological response of the organism to the implant, especially immediately after the implantation procedure. However, the zeta potential method used in these in vitro studies could not be used in vivo due to technical limitations. Moreover, determination of the breakdown potential of the 2G ONTs layer on the Ti–13Zr–13Nb alloy in PBS was not possible due to the technical limitations of the potentiostat to the tested potential range of 10 V. 

The conducted research encourages further research on surface modification of the biomedical Ti–13Zr–13Nb alloy by anodizing in new electrochemical conditions. In the near future, in vitro and in vivo biological tests are planned, which will make it possible to obtain the CE certificate required to implement the developed surface modification technology. Knowledge about the kinetics of drug release from the obtained ONTs will facilitate the future development of personalized implants that are carriers of tissue-forming and therapeutic substances, supporting the process of osseointegration of the implant in the human body.

## Data Availability

Not applicable.
